# Critical Needs and Opportunities for Advanced Manufacturing of Lyophilized Injectables

**DOI:** 10.1007/s11095-025-03869-5

**Published:** 2025-06-27

**Authors:** Alina Alexeenko, Maxwell Korang-Yeboah, Serguei Tchessalov

**Affiliations:** 1 Purdue University, West Lafayette, IN 47907 USA; 2 Office of Pharmaceutical Quality Research, CDER, FDA, Silver Spring, MD 20993 USA; 3 Biotherapeutics, Pharmaceutical Sciences, Pfizer Inc., Andover, MA 01810 USA

**Keywords:** Advanced manufacturing technology, Freeze-drying, Lyophilization

## Abstract

Lyophilized drugs and biologics have an outsized role in protecting public health due to their ability to provide extended shelf life for stockpiling. Over 70% (14 out of 19) of antibiotics on the Essential Medicines list are supplied as lyophilized sterile powders for injection (FDA, 1). Additionally, many new drugs, including first-in-kind medicines, such as the first checkpoint inhibitor cancer immunotherapy Keytruda, were initially introduced to market in a lyophilized form, accelerating availability to patients by several years while a stable liquid formulation was being developed. The article describes methodologies, both short-term and long-term, to address the current manufacturing challenges for lyophilized injectables based on the findings of the workshop by National Institute of Pharmaceutical Technology and Education held in January 2024.

The number of FDA approvals for lyophilized injectables (Fig. 1) has increased by over 300% since 2000s. Most lyophilized medicines are generic small molecule drugs (70% in 2022). While the overall demand for lyophilization capacity has increased, generics manufacturers often use legacy processes approved 20 years ago due to low incentives and high risks to modernize the manufacturing equipment and processes. Currently, the global production capacity can be described as strained, with lyophilized injectables comprising 13% of all products on the FDA’s “in shortage” list in 2021. In a recent example, Covid-19 vaccines had to be frozen because of lack of total lyophilization capacity to deliver billions of doses within a few months.

**Fig. 1:**
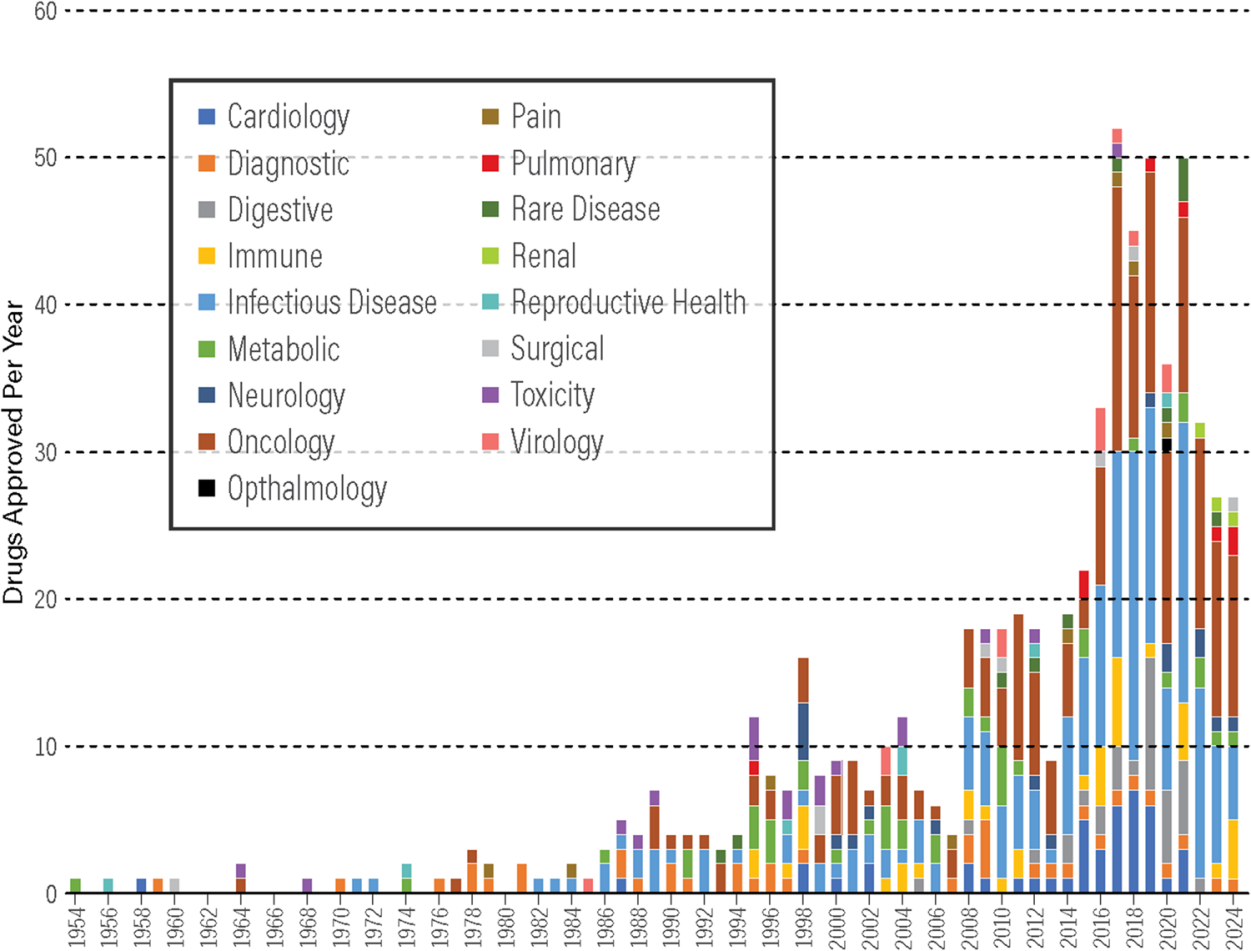
Annual approvals by FDA of lyophilized drugs and biologics. Source of data: LyoHUB Lyo Drug Database

Overall, major manufacturers are faced with the need to expand production. A 2024 LyoHUB member survey, involving a mix of large manufacturers and CDMOs, showed an average increase in capacity of ~ 100% since 2010 with 5 out of 8 manufacturers responding that they are currently in the process of further expanding or planning to expand lyophilization capacity. The build-up provides an opportunity to adopt better manufacturing practices to strengthen the supply chain of injectable medicines. Based on discussions at the 2024 NIPTE Injectables workshop, we provide here a summary of practices that could be rapidly deployed for improving lyophilization throughput and yield (“small steps”) and emerging technologies that have a promise of revolutionary advancements (“giant leaps”) in capability to supply lyophilized injectables. Small steps center on increased efficiency and robustness of established lyophilization techniques, whereas giant leaps enable previously unattainable speed and scale through integration of new technological approaches.

## Small Steps: Understand Failure Modes of Product and Equipment to Proactively Optimize Performance and Mitigate Risks

The inability to maintain manufacturing quality is one of the biggest causes of supply chain disruption [2]. Disruptions to manufacturing operations due to equipment failure may have ripple effect on the drug supply chain especially for lyophilized injectable generic drug products. Thus, there is the need for appropriate risk mitigation strategies to ensure such disruptions to manufacturing operations are minimized. A fundamental component of quality risk management is the identification of potential failure modes of the equipment and understanding of the associated risk to product quality. Further systematic characterization of the lyophilizer capability, performance, and product failure modes may offer the added advantage of expansion of the operational design space of the lyophilization process and ultimately improve efficiency and robustness.

Despite the importance of lyophilization, a recent survey [3] revealed inadequate characterization of products and equipment prior to process design. Specifically critical product temperatures, specifically Tg’ and T_collapse_ measured using DSC and FDM [4] are not always obtained prior to lyophilization process development. In addition, equipment characterization data are inadequate to demonstrate the lyophilization process may consistently yield products of the required quality. Key equipment performance characteristics [5] such as shelf temperature mapping with load, the minimum controllable pressure and maximum sublimation rate may not always be measured. The inadequate product and process characterization may be more common in legacy products approved prior to advances in analytical equipment for product characterization and sensors for process monitoring. This leads to suboptimal processes and limits the capability to improve the performance of currently inefficient legacy processes. Thus, for legacy lyophilized injectables in shortage or at risk of shortage, systematic characterization of lyophilizer capability and performance, and product failure modes could be an important step towards improving process efficiency and robustness.

Monitoring and detection of signals of equipment failure is another advancement that may reduce disruptions to manufacturing of lyophilized injectables. Thus, proactive monitoring and detection of signals of equipment failure can be a part of the overall risk management strategy. Lyophilization is a data rich process that eases implementation of proactive data driven quality risk management practices. Such practices may take the form of simple historical, time series, and trend analysis or advanced AI/ML models of equipment data for proactively gaining insights into the overall health of the manufacturing process. For example, safe yet observable variations in condenser coil temperature and lyophilizer evacuation time have been shown to provide early signals of equipment failure.

The value of real time monitoring and control of pharmaceutical processes at all scale cannot be overstated. Yet, there is limited adoption of process analytical technologies for manufacturing of lyophilized injectables at commercial scale, especially for generic drug products. Comparative pressure monitoring of the chamber and condenser is another advancement with little barrier to implementation with considerable upside. In comparative pressure monitoring, both a Pirani gauge and capacitance monometer have been used for monitoring the pressure in the chamber and condenser of the lyophilizer [6]. The monitoring has been shown to provide accurate determination of the end point of primary drying, allowing avoidance of some of the most common lyophilization-related process deficiencies [7]. The pressure monitoring technique takes advantage of the difference in the mechanism of pressure measurement by the Capacitance manometer and Pirani gauges for real time monitoring and control of the primary and secondary drying steps. Further having both gauges on the condenser allows equipment capability assessment specifically (i.e., choke flow measurement), real time assessment of equipment performance and troubleshooting of equipment failure (i.e., leak source detection). The technique of pressure monitoring is inexpensive and has been proven to be sensitive, reliable, and robust and requires little change to current processes and equipment. Recommended methods for leak monitoring and acceptable leak rates have been published by [8] based on a survey conducted by BioPhorum. Further the implementation of comparative pressure measurement at commercial scale may ease the development and adoption of process models (see commentary on process modeling below) and the transition of the lyophilization process to the next phase of industrial revolution. An industry led guidance on use of pressure sensors was proposed in the best practices paper [9], yet implementation of this PAT tool is still very slow.

It is not unusual that, after multiple steam sterilization cycles and shelf movements for stoppering of vials, lyophilizer hoses become fragile allowing leaks of heat transfer fluid (HTF) and contamination of drug products. Unfortunately, it is impossible to salvage the drug products upon detection of HTF in the product during testing. Residual gas analysis of the equipment prior to lyophilization allows early detection of HTF leakage and prevention of potential product contamination. Residual gas analyzers (RGA) also provide useful information on changes in gas composition during drying and can be used for process monitoring and leak detection [10]. RGA is a mass spectrometry-based technique that measures the partial pressure of gas species in a vacuum system. They are small, relatively low-cost devices (a typical RGA costs a fraction of a laboratory-scale lyophilizer and ~ 1% of production-scale equipment) that are easy to install on existing lyophilizers. Implementation of RGA on the commercial scale, however, is still slow partly because of sensitivity of RGA to silicone oil contamination. Recent advances in RGA design with a silicone oil filter [11] could potentially increase life span of RGA sensors. Other methods of avoiding failure of lyophilization equipment involve monitoring condenser coil temperature and its drift over time from batch to batch, time to pump to a certain pressure, and shelf temperature and chamber pressure control.

The freezing step of conventional lyophilization process involves decreasing and holding the shelf temperature below the glass transition temperature of the freeze concentrate until complete solidification. This process is associated with random ice nucleation and a higher degree of supercooling that subsequently can result in an inefficient process and higher heterogeneity in product quality attributes. Technological advancement has allowed the control of the ice nucleation (CIN) temperature and reduction of the degree of supercooling during the freezing step. Several groups have shown adoption of controlled ice nucleation technology led to improvement in process efficiency and robustness. Further, CIN has the added advantage of improving product quality attributes such as reconstitution time, appearance, aggregation rate, while reducing inter vial variability in product quality attributes. Despite the advantages, only a handful of FDA approved products employs CIN. The slow adoption of CIN may be attributed to perceived scientific, technical, and regulatory challenges. CIN technologies rapid pressurization-depressurization (ControLyo®) and ice fog (i.e., VERISEQ®) are available at commercial scale. Depending on the technology adopted, minimal changes to existing lyophilizers may be required. Although the impact of CIN on process efficiency may be most important in a few product categories such as those with low temperature collapse, high fill volume, a high concentration of biologic dug substance or surface sensitive products, the overall benefit of adoption of CIN may outweigh the risks. Further the regulatory barrier for adoption for CIN may be lower compared to the past as the technology has been approved for commercial manufacturing (albeit for only a few products). Thus, CIN is a step with potential substantial upside for the manufacturing of lyophilized injectables.

The use of process models enhance process understanding, reduce the number of experimental studies required to design a robust and efficient process and ultimately saves cost. Use of simple quasi-steady vial heat and mass transfer models known as “lyocalculator” can help avoid costly trial and error in process development [12], scale up and heterogeneity analysis [13]. Yet the adoption of process modeling at commercial scale appears to be limited which is attributed to the low adoption of PAT at commercial scale as well as development of heat, K_v_, and mass, R_p_, transfer parameters. Recent work [14] at Sanofi demonstrated the feasibility of using traditional equipment parameters such as pressure differences between chamber and condenser as well as temperature differences between inlet and outlet of the heat transfer fluid for estimating the sublimation flow rate during primary drying and subsequently the average product temperature. The approach introduced by the Sanofi’s team combined with modeling of primary drying provides practical real-time monitoring of lyophilization rate. Further, this could be used for close-loop process control and performance optimization.

A critical step towards understanding failure modes and mitigating risks of lyophilization involves better lyophilization process understanding through education and training. Despite growing demands in lyophilized products, to the best of our knowledge no undergraduate programs in the US currently teach lyophilization as part of required curriculum in chemical engineering or pharmaceutical sciences, perhaps due to complexity of the process. Existing graduate programs developed in active research centers on lyophilization do not produce a critical workforce mass to cover current and future needs. Increased access to lyophilization education can be provided through low-cost online curricula and hands-on training in shared facilities open to non-degree-seeking students such as operators and industry professionals.

## Giant Leaps: Adopting Alternative Aseptic Drying Technologies

Conventional batch vial freeze-drying process has many disadvantages which results in long and expensive processes, including: heterogeneity during drying, inefficient process control and lack of tools to measure product critical attributes during cycle. Recently, a few alternative new technologies matured enough to overcome these challenges and produce shelf-stable easy-to-reconstitute dry dosage forms. Notable approaches applicable to manufacturing of dry injectables are bulk spray freeze-drying, spin freeze-drying, spray drying, and microwave vacuum drying, all of which can be done in aseptic manner. Fundamentally, these techniques are utilizing a combination of more effective heat transfer, by forced convection or electromagnetic radiation instead of gas conduction, and less resistance to mass transfer by decreasing the size of the dry product layer. The more effective heat transfer also alleviates the common “edge effect” problem of conventional freeze-drying in tray-style lyophilizers.

- Spray freeze-drying (SFD) is well known process which, until now, did not find a proper application in commonly used commercial processes for the temperature sensitive injectables. Introduction of new nozzle designs and double wall columns allow aseptic manufacture of pellets with narrow size distribution (~ 0.5 mm in diameter) that, once dried, have excellent flowability [15, 16]. The sizes of the pellets are small enough to allow fast drying (about 2 h for a single layer of pellets) but large enough to enable seamless powder filling with accuracy better than 2%. Another benefit of small size is the ability to dry products exhibiting very low collapse temperatures (T_c_ < −40 °C) in a reasonable time (~ 10 h). As compared to vial freeze-drying processes, spray freeze-drying could produce, on average, twice as much product per sq.m of manufacturing area and, the product produced per sq.m. would be even more pronounced for the low collapse products which have longer cycle times. Once the bulk of dried pellets is available, the pellets can be filled in any type of container (dual chamber syringes/cartridges, bottles, bags etc.). It has been shown that spray freeze-dried products reconstitutes faster than the same amount of material dried in a vial conventionally. SFD could potentially reduce use of electricity, water for injection, steam, and liquid nitrogen by more than 70% to provide a sustainable attractive solution for the future drying equipment [17]. As of now, the process can be implemented aseptically in batch by rotary drying or continuously. The significant capital cost, however, prevents companies investing in this technology given that large pharmaceutical companies currently have a significant number of conventional freeze-dryers. Combining efforts from the different companies and building together pilot/commercial scale spray freeze-dryer and powder filling machine could be the first step in implementation of this technology.
- Aseptic spray drying is already in use for manufacture of small-molecule drugs (Arpagaus, 2023) with cGMP systems available from multiple vendors (e.g. GEA Niro or SPX). In 2015 FDA approved Raplixa™, the first biopharmaceutical manufactured using an aseptic spray drying. This paved a pathway for aseptic spray drying and aseptic spray freeze-drying as alternatives to lyophilization for manufacturing of biologics. The basic components of aseptic spray drying systems are similar to regular spray drying and include spray atomization, drying chamber with heated sterile gas flow, cyclone collection, and filtration. Additional aseptic components include HEPA filtration for the drying gas and clean-in-place and sterilize-in-place systems as well as sterile powder handling. The throughput of aseptic spray drying can be significantly higher than a conventional freeze-drying with a smaller footprint. Additionally, spray drying does not require vacuum and refrigerant handling systems and less prone to associated failure of equipment due to leaks. However, aseptic spray drying requires additional sterile powder filling systems. Powder filling is typically associated with increased losses and lower rates as compared with liquid fill that is used in conjunction with conventional freeze-drying in containers. The scale up from development to manufacturing for aseptic spray drying is not straightforward which may present challenges for formulation and process development for spray drying of sterile injectables, especially for higher cost biologics.
- Spin freeze-drying presents a continuous freeze-drying technology [18] based on spinning the vials while cooling and freezing by cold gas, resulting in a thin product layer (1–1.2 mm) spread over the inner vial wall. The spin frozen vials are subsequently dried under vacuum, where individual infrared heaters provide the radiative energy transfer for ice sublimation. Besides faster drying – up to 10–40 times, depending on the specific formulation properties and vial dimensions [19] – due to large sublimation surface and thin product layer, the technology could offer accurate product temperature control for every single vial, thereby reducing the heterogeneity of the lyophilization process. The energy consumption could also be reduced by factor of 2. Because spin freeze drying is a closed process, the requirements to maintaining grade A area are greatly reduced and that space saving is reflected in the total cost of manufacturing. Relatively low throughput, as compared to the existing large scale lyophilizers, directs the use of this technology towards novel small-batch, high value temperature sensitive products such as gene therapies and antibody drug conjugates. Implementation of this technology is currently slow, and is likely linked to the slow introduction of new, dried modalities to the market as opposed to their liquid or frozen counterparts.
- Microwave vacuum drying refers to the use of non-ionizing electromagnetic energy in the microwave frequency range applied to frozen materials under vacuum conditions (< 500 mtorr). A common form of MVD such as developed by EnWave uses passthrough microwave sources at 2.45 GHz with wide availability of power components at this frequency band for industrial and scientific use. MVD has been demonstrated an increase in the drying rates by > 5 × over traditional freeze-drying times while maintaining comparable product activity and stability for biologics [20]. Recent advances in solid-state RF power sources for satellite communication at the higher frequency in X and Ku bands provided compact energy sources with more effective heating of ice and better uniformity [21]. Such microwave sources incorporated into traditional freeze-drying and alternative drying systems provide volumetric heating with fast response times compared to heat transfer fluids as in traditional freeze-drying and aseptic drying. Scalability of this approach for cGMP needs to be evaluated.

In summary, significant efforts are being made across the industry and academia to tackle process heterogeneity and lengthy cycle times in pharmaceutical lyophilization. Key enablers include PAT tools, modeling and simulation, improved equipment, and broad participation in technology demonstrations with regulatory support for accelerated adoption of new technologies to meet the growing demand for lyophilized injectable drug products and biologics.
